# Changes in the prevalence of severe anxiety and depression symptoms and the associated factors in adults living in Manaus: a comparison of two cross-sectional studies conducted in 2015 and 2019

**DOI:** 10.1590/1516-3180.2023.0424.R1.03072024

**Published:** 2024-12-20

**Authors:** Vanessa Gomes Lima, Marcus Tolentino Silva, Gustavo Magno Baldin Tiguman, Taís Freire Galvão

**Affiliations:** I Graduate Program of Pharmaceutical Sciences, School of Pharmaceutical Sciences, Universidade Estadual de Campinas (UNICAMP), Campinas (SP), Brazil.; II Professor, Department of Public Health, School of Health Sciences, Universidade de Brasília (UNB), Brasília (DF), Brazil.; III Graduate Program of Pharmaceutical Sciences, School of Pharmaceutical Sciences, Universidade Estadual de Campinas (UNICAMP), Campinas (SP), Brazil.; IV Graduate Program of Pharmaceutical Sciences, School of Pharmaceutical Sciences, Universidade Estadual de Campinas (UNICAMP), Campinas (SP), Brazil.

**Keywords:** Anxiety., Depression., Prevalence., Socioeconomic factors., Cross-sectional studies., Mental health., Mental suffering., Austerity., Survey., Amazonas., Brazil.

## Abstract

**BACKGROUND:**

Emotional distress increases, also affected by the setting.

**OBJECTIVE:**

To estimate changes in prevalence of severe anxiety and depressive symptoms and associated factors.

**DESIGN AND SETTING:**

This cross-sectional study included adults living in Manaus selected through a three-stage probability sampling in 2015 and 2019.

**METHODS:**

This is an analysis of two surveys conducted. The outcomes were assessed by Generalized Anxiety Disorder 7-item (≥ 15 points) and Patient Health Questionnaire 9-item (≥ 20), and changes were tested by chi-square goodness-of-fit. Prevalence ratios (PR) with 95% confidence intervals (95%CI) were calculated by Poisson regression.

**RESULTS:**

Severe anxiety symptoms increased from 3.3% (95%CI = 2.7–3.9) in 2015 (n = 3,479) to 8.7% (95%CI = 7.5–9.8) in 2019 (n = 2,321); severe depressive symptoms changed from 2.5% (95%CI = 2.0–3.0) to 8.5% (95%CI = 7.3–9.6). Variations were more pronounced in social vulnerability (P < 0.05). Outcomes were higher in women (anxiety: PR = 1.27; 95%CI = 1.20–1.34, depression: PR = 1.35; 95%CI = 1.27–1.44), low-income individuals (anxiety: PR = 1.90; 95%CI = 1.20–3.00, depression: PR = 1.98; 95%CI = 1.22–3.19), less educated individuals (anxiety: PR = 2.20; 95%CI = 1.16–4.18, depression: PR = 2.37; 95%CI = 1.23–4.60), and individuals with poor health status (anxiety: PR = 9.06; 95%CI = 6.72–12.21, depression: PR = 8.99; 95%CI = 6.67–12.12).

**CONCLUSION:**

Severe anxiety and depression tripled in Manaus, potentially reflecting Brazilian socioeconomic crises.

## INTRODUCTION

Mental health is influenced by social, economic, cultural, racial, psychological, and behavioral factors, which mediate suffering and can trigger disorders such as anxiety and depression.^1^ From the onset of symptoms to the treatment of these conditions, individuals encounter numerous challenges, including limited access to specialists, a constrained therapeutic arsenal, and inadequate social care. These barriers can contribute to the chronicity of symptoms associated with anxiety and depression.^2^

The global prevalence of affective disorders is rising, with depressive states increasing by approximately 50% since the 1990s, predominantly affecting women.^3^ In Brazil, depressive symptoms are associated with a high prevalence of non-communicable chronic diseases, as the daily limitations imposed by chronically ill exacerbate mental suffering, leading to depressive symptoms.^4^

Since 2016, Brazil has been experiencing increases in unemployment rate, income inequality, and reductions in public support for basic social programs due to ongoing economic and political crises.^5^ These measures included reforms in the healthcare system and reductions in social spending.^6^ In this social insecurity setting, stress and fear, among other feelings of restlessness, impair mental health at the individual and collective levels.^7^

The Brazilian Amazon is one of the country’s least developed regions, with significant income inequality and limited research on mental health and its social effects.^8^ In 2015, a survey was carried out in the Manaus Metropolitan Region.^9^ In 2019, another population-based survey was conducted exclusively in Manaus.^10^ Anxiety and depressive symptoms affected over 20% of *Manauaras* adults in 2019.^11^ Meanwhile, the prevalence was < 10% in the whole metropolitan area in 2015,^12,13^ indicating an increase in mental suffering in this population. Comparing the population of the same city in these two periods would help identify the predictors of mental health disorders and assess the effects of the social context in the Brazilian Amazon.

## OBJECTIVE

This study aimed to estimate the changes in the prevalence of severe anxiety and depressive symptoms from 2015 to 2019 and identify the factors associated with severe symptomatology among adults living in Manaus.

## METHODS

### Study design

Two cross-sectional population-based studies conducted in adults (≥ 18 years old) living in Manaus, Brazil, in 2015 and 2019 were analyzed. The 2015 survey examined the metropolitan area of Manaus and seven other cities. In the present analysis, the sample was restricted to adults residing in Manaus to enable comparison with the results of the 2019 survey, which focused exclusively in the capital.^14^

### Setting

This study was conducted in Manaus, the capital of the state of Amazonas, which is the most economically and densely populated city in the state, housing over half of its inhabitants in 2018. In the same year, the state ranked fourth in terms of income inequality (Gini index 0.523) and had a high percentage of public healthcare demand (84%).^14^ Manaus is one of the Brazilian cities with the largest gross domestic product (78 billion Brazilian reais, accounting for 1% of the gross national product in 2018). However, this wealth is unevenly distributed, resulting in significant social inequalities.^14^

### Participants

Probabilistic sampling was conducted in three stages to select participants for both surveys. In the first stage, census tracts were randomly selected. In the second stage, households were chosen through systematic sampling. A number from 1 to 20 was randomly assigned to determine the first household to be visited, ensuring that 1 out of every 20 households was visited. All residents present in the household were registered in the electronic devices used for the interview. One participant was selected based on predefined quotas for age and sex according to the proportions estimated by the Brazilian Institute of Geography and Statistics for each time point to ensure population representativeness.^9,10^

### Variables

The primary outcomes were the prevalence and severity of anxiety and depressive symptoms. The independent variables were sex (men or women), pregnancy (yes or no), age (18–24, 25–34, 35–44, 45–59, or ≥ 60 years), ethnicity (White [White and Asian] or Black [Black, Brown, and Indigenous]), the presence of a partner (yes or no), education (higher education or above, high school, elementary school, or less than elementary school), occupation (formal worker, informal worker, retired, student, or unemployed/housewife), social class (A/B, C, or D/E, where A refers to the wealthiest and E refers to the poorest based on the Brazilian Economic Classification Criteria of each year),^15,16^ and self-reported health status (good, fair, or poor).

### Data sources and measurement

A team of trained and experienced interviewers collected data from the participants using questionnaires preconfigured in the SurveyToGo software, with the aid of electronic devices (Tab3 SM-T110 Samsung^®^ Galaxy, in 2015 and Intel TabPhone 710 Pro, in 2019). Data were collected offline and subsequently transmitted to a research database via the Internet.

Anxiety symptoms were assessed using the validated version of the Generalized Anxiety Disorder 7-item (GAD-7).^17,18^ The questionnaire comprises seven items that assess the symptoms observed in the last two weeks, with a total score ranging from 0 to 21. The anxiety symptoms were categorized as minimal or none (0–4), mild (5–9), moderate (10–14), or severe (15–21).^19^ A cutoff value of ≥ 10 points was used to indicate the presence of anxiety symptoms, with sensitivity and specificity greater than 80% compared with the mental health professionals’ clinical diagnosis. Severe anxiety was defined as a GAD-7 score of ≥ 15.^20^

The validated version of the nine-item Patient Health Questionnaire 9-item (PHQ-9) was used to assess depressive symptoms.^21^ Based on the instrument’s nine questions, depressive symptoms were categorized as minimal or none (0–4), mild (5–9), moderate (10–14), moderately severe (15–19), or severe (20–27), with the total scores ranging from 0 to 27. A score of ≥ 10 points indicated the presence of depressive symptoms, with a sensitivity of 78% and a specificity of 87% (compared to the clinical diagnosis made by a psychiatrist).^22^ Severe depressive symptoms were considered present if the final score of PHQ-9 was ≥ 20.^23^

### Bias

To avoid biases related to the research instrument, several precautions were taken. The data were automatically tabulated by transmitting the questionnaires completed on the tablets to the online database. Face-to-face interviews were conducted using validated instruments to measure the main outcomes and variables, which increased the response rate and data reliability. The questionnaire was pre-tested with 150 participants from various social levels to ensure comprehension. To allow reliability of data collection, part of interviews were audio recorded by the electronic device and 20% of interviews were audited by telephone.

### Study size

The sample size was determined based on the population estimates for each period. In the 2015 survey, 4,000 of the 2,106,322 adult inhabitants were planned to be interviewed across the Manaus Metropolitan Region. This estimate was based on an anticipated 50% healthcare service usage, 95% confidence level, 2% absolute accuracy, and a design effect of 1.5.^9^ In 2019, 2,300 interviews were planned considering the results of the previous study, which found that 20% of the participants reported seeking health services in the last 15 days, 2,145,444 adults living in Manaus and similar parameters to the previous survey.^10^

### Statistical methods

The prevalence of severe anxiety and depressive symptoms and the corresponding 95% confidence intervals (95%CI) were estimated and described according to the independent variables. The differences in absolute (Δ) and relative (ratio) frequencies from 2015 to 2019 were calculated, and significance was assessed using the chi-square goodness-of-fit test. The outcomes were stratified by year, sex, and health status, and the Pearson’s correlation coefficient (r) was estimated to examine the relationship between the GAD-7 and PHQ-9 scores.

A Poisson regression with robust variance was performed to assess the factors associated with severe anxiety and depressive symptoms. Prevalence ratios (PR) and 95%CI of severe anxiety and depression symptoms were calculated and adjusted for year of research, sex, and age, in separate models. All analyses employed a complex sampling design (*svy* command) and were performed using the Stata statistical package (version 12.4).

### Ethics

Both surveys were approved by the Universidade Federal do Amazonas Research Ethics Committee (opinion letter No. 974,428 of March 3, 2015, and Certificate of Presentation for Ethical Assessment [CAAE]: 42203615.4.0000.5020; 2019: opinion letter No. 3,102,942 on December 28, 2018, and CAAE: 04728918.0.0000.5020). All participants signed an informed consent form prior to the interviews.

## RESULTS

**Figure 1:**
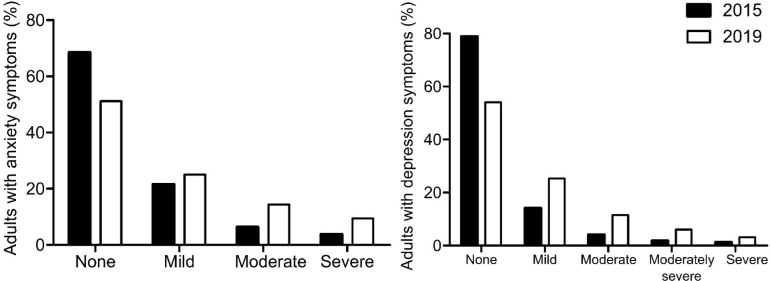
Distribution of anxiety and depression symptoms in adults living in Manaus, according to the severity, in 2015 (n = 3,479) and 2019 (n = 2,321).

We included 5,800 participants (3,479 in 2015 and 2,321 in 2019). The prevalence of severe anxiety symptoms were 3.3% (95%CI = 2.7%–3.9%) in 2015 and 8.7% (95%CI = 7.5%–9.8%) in 2019. Similarly, the prevalence of severe depressive symptoms increased from 2.5% (95%CI = 2.0%–3.0%) in 2015 to 8.5% (95%CI = 7.3%–9.6%) in 2019. Comparisons between the 2019 with 2015 survey results suggested a relative increase in the prevalence of both severe anxiety and depressive symptoms (**Figure 1**).

The prevalence of severe anxiety symptoms was higher in women (4.8% in 2015 and 11.6% in 2019), those belonging to the lower social classes (4.3% in 2015 and 9.6% in 2019), and those with poor health (16.7% in 2015 and 31.7% in 2019). Severe depressive symptoms were more frequent in women (4.2% in 2015; 11.0% in 2019), individuals from the lower social classes (3.7% in 2015; 9.6% in 2019), and individuals with poor health (13.9% in 2015; 31.4% in 2019) (**Table 1**). Higher absolute changes (Δ) in the prevalence of severe anxiety and depressive symptoms were observed among women (anxiety and depression: 6.8%), individuals with lower educational levels (5.9% and 7.3%, respectively), and those with poor health status (15.0% and 17.5%). Conversely, the highest ratio of prevalence between 2015 and 2019 was observed in the youngest adults (5.8 and 6.6; **Table 1**). The prevalence rates of anxiety symptoms (of any severity) were 9.1% (95%CI = 8.2%–10.1%) in 2015 and 22.4% (95%CI = 20.7%–24.1%), in 2019. Meanwhile, depressive symptoms affected 6.3% (95%CI = 5.5%–7.1%) of the adults in 2015 and 19.9% (95%CI = 18.2%–21.5%) in 2019.

**Table 1:** Main characteristics of the participants, prevalence, absolute and relative differences of severe anxiety and depression symptoms in Manaus, 2015 (n = 3,479) and 2019 (n = 2,321)

Variables	Total, n (%)		Severe anxiety symptoms, n (%)				Severe depressive symptoms, n (%)			
	2015	2019	2015	2019	Δ	Ratio	2015	2019	Δ	Ratio
**Sex**										
Men	1,623 (47.9)	1,088 (47.8)	26 (1.6)	59 (5.4)	3.8	3.4	11 (0.7)	61 (5.6)	5.0	8.4
Women	1,856 (52.2)	1,233 (52.2)	88 (4.8)	144 (11.6)	6.8	2.4	77 (4.2)	137 (11.0)	6.8	2.6
**Pregnant**										
No	1,657 (89.9)	1,091 (88.3)	79 (4.9)	131 (11.9)	7.1	2.4	66 (4.1)	125 (11.4)	7.3	2.8
Yes	199 (10.1)	142 (11.7)	9 (4.5)	13 (9.3)	4.8	2.1	11 (5.5)	12 (8.4)	2.9	1.5
**Age group (years)**										
18–24	716 (19.6)	405 (19.3)	11 (1.5)	35 (8.5)	7.0	5.8	10 (1.4)	37 (9.0)	7.7	6.6
25–34	1,010 (27.5)	586 (25.1)	29 (2.8)	41 (7.0)	4.2	2.5	28 (2.7)	44 (7.5)	4.8	2.8
35–44	744 (22.4)	553 (22.9)	24 (3.2)	53 (9.5)	6.3	3.0	13 (1.7)	52 (9.3)	7.6	5.4
45–59	674 (20.2)	526 (21.2)	31 (4.6)	53 (10.0)	5.4	2.2	24 (3.6)	48 (9.1)	5.5	2.5
≥ 60	335 (10.3)	251 (11.6)	19 (5.7)	21 (8.4)	2.8	1.5	13 (3.9)	17 (6.8)	2.9	1.8
**Ethnicity**										
White	674 (19.3)	349 (15.1)	19 (2.8)	34 (9.6)	6.8	3.4	17 (2.5)	28 (8.0)	5.5	3.2
Black	2,805 (80.7)	1,972 (85.0)	95 (3.4)	169 (8.5)	5.1	2.5	71 (2.5)	170 (8.5)	6.0	3.4
**Marital status**										
Without partner	1,636 (47.0)	1,005 (44.1)	43 (2.7)	89 (8.7)	6.0	3.2	40 (2.5)	99 (9.7)	7.2	4.0
With partner	1,843 (53.0)	1,316 (56.0)	71 (3.8)	114 (8.6)	4.8	2.2	48 (2.6)	99 (7.5)	4.9	2.9
**Educational level**										
Higher education or above	131 (3.8)	153 (6.5)	2 (1.5)	8 (5.3)	3.8	3.5	3 (2.2)	7 (4.7)	2.5	2.2
High school	1,695 (48.3)	1,171 (50.4)	37 (2.2)	91 (7.7)	5.5	3.5	28 (1.6)	89 (7.6)	5.9	4.6
Elementary school	562 (16.0)	432 (18.9)	20 (3.6)	41 (9.4)	5.8	2.6	16 (2.8)	38 (8.8)	6.0	3.1
Less than elementary	1,091 (31.9)	565 (24.2)	55 (5.0)	63 (10.9)	5.9	2.2	41 (3.8)	64 (11.1)	7.3	2.9
**Economic classification**										
A/B	555 (16.0)	282 (12.2)	8 (1.4)	14 (5.0)	3.6	3.5	6 (1.0)	14 (5.0)	3.9	4.8
C	2,006 (57.4)	1,244 (53.6)	66 (3.4)	112 (8.9)	5.6	2.7	47 (2.4)	106 (8.5)	6.1	3.6
D/E	918 (26.5)	795 (34.1)	40 (4.3)	77 (9.6)	5.2	2.2	35 (3.7)	78 (9.6)	5.9	2.6
**Occupation**										
Formal job	651 (18.8)	419 (17.9)	13 (2.0)	36 (8.6)	6.6	4.3	7 (1.1)	30 (7.2)	6.1	6.7
Informal job	978 (28.5)	661 (28.1)	27 (2.7)	39 (5.8)	3.1	2.2	18 (1.8)	48 (7.3)	5.5	4.1
Retired	271 (8.2)	162 (7.2)	20 (7.5)	18 (10.9)	3.5	1.5	14 (5.2)	13 (7.8)	2.6	1.5
Student	315 (8.7)	124 (5.7)	5 (1.6)	9 (7.1)	5.5	4.5	5 (1.5)	9 (7.2)	5.6	4.7
Unemployed/housewife	1,264 (35.7)	955 (41.0)	49 (3.9)	101 (11.0)	7.0	2.8	44 (3.5)	98 (10.2)	6.6	2.9
**Health status**										
Good	2,243 (64.1)	1,498 (64.8)	33 (1.5)	53 (3.5)	2.0	2.4	21 (0.9)	60 (4.0)	3.1	4.5
Fair	1,012 (29.3)	671 (28.8)	44 (4.3)	102 (15.1)	10.9	3.5	36 (3.5)	90 (13.3)	9.8	3.8
Poor	224 (6.6)	152 (6.5)	37 (16.7)	48 (31.7)	15.0	1.9	31 (13.9)	48 (31.4)	17.5	2.3
**Total**	3,479 (100)	2,321 (100)	114 (22.5)	203 (50.3)	27.9	7.8	88 (18.3)	198 (48.7)	30.4	10.6

After adjustment, the prevalence of severe anxiety and depressive symptoms was significantly higher in 2019 in women (anxiety: PR = 1.27; 95%CI = 1.20–1.34, depression: PR = 1.35; 95%CI = 1.27–1.44), individuals with lower income (anxiety: PR = 1.90; 95%CI=1.20–3.00, depression: PR = 1.98; 95%CI = 1.22–3.19), and less educated individuals (anxiety: PR = 2.20; 95%CI = 1.16–4.18, depression: PR = 2.37; 95%CI = 1.23–4.60), and in those with worse health status (anxiety: PR = 9.06; 95%CI = 6.72-12.21, depression: PR = 8.99; 95%CI = 6.67–12.12) (**Table 2**). The prevalence of severe anxiety was also higher in individuals who only finished elementary education (PR = 1.96; 95%CI = 1.01–3.79) compared with those who achieved a higher education. Age, pregnancy in the previous year, ethnicity, the presence of a partner, and occupation were not associated with the outcomes (**Table 2**).

**Table 2:** Unadjusted and adjusted prevalence ratios (PR) with 95% confidence intervals (95%CI) of severe anxiety and depression symptoms by independent variables

Variables	Severe anxiety symptoms				Severe depressive symptoms			
	PR unadjusted (95%CI)	P value	PR adjusted (95%CI)	P value	PR unadjusted (95%CI)	P value	PR adjusted (95%CI)	P value
**Year**								
2015	1.00	< 0.001	1.00	< 0.001	1.00	< 0.001	1.00	< 0.001
2019	2.63 (2.09–3.28)		1.27 (1.20–1.34)		3.35 (2.62–4.30)		1.35 (1.27–1.44)	
**Age group (years)**								
18–24	1.00	0.045	1.00	0.094	1.00	0.733	1.00	0.733
25–34	0.95 (0.66–1.39)		0.96 (0.66–1.39)		0.95 (0.66–1.38)		0.96 (0.67–1.38)	
35–44	1.27 (0.89–1.83)		1.25 (0.87–1.78)		1.07 (0.74–1.56)		1.05 (0.73–1.51)	
45–59	1.46 (1.02–2.08)		1.42 (1.00–2.02)		1.22 (0.85–1.75)		1.18 (0.82–1.69)	
≥ 60	1.40 (0.92–2.14)		1.30 (0.85–1.97)		1.03 (0.65–1.62)		0.94 (0.60–1.49)	
**Sex**								
Men	1.00	< 0.001	1.00	< 0.001	1.00	< 0.001	1.00	< 0.001
Women	2.32 (1.81–2.98)		2.31 (1.81–2.95)		2.38 (1.83–3.09)		2.38 (1.83–3.09)	
**Pregnant**								
No	1.00	0.431	1.00	0.905	1.00	0.661	1.00	0.940
Yes	0.84 (0.54–1.30)		0.97 (0.62–1.53)		0.91 (0.59–1.40)		0.98 (0.62–1.55)	
**Ethnicity**								
White	1.00	0.862	1.00	0.767	1.00	0.401	1.00	0.815
Black	1.03 (0.77–1.38)		0.96 (0.72–1.28)		1.15 (0.83–1.58)		1.04 (0.76–1.42)	
**Marital status**								
Without partner	1.00	0.336	1.00	0.747	1.00	0.178	1.00	0.045
With partner	1.12 (0.89–1.39)		1.04 (0.83–1.30)		0.85 (0.68–1.07)		0.78 (0.62–0.99)	
**Educational level**								
Higher education or above	1.00	0.006	1.00	0.007	1.00	0.014	1.00	0.002
High school	1.29 (0.68–2.45)		1.51 (0.80–2.84)		1.27 (0.66–2.42)		1.48 (0.78–2.82)	
Elementary school	1.71 (0.88–3.33)		1.96 (1.01–3.79)		1.64 (0.83–3.22)		1.85 (0.94–3.63)	
Less than elementary	1.93 (1.02–3.66)		2.20 (1.16–4.18)		1.87 (0.97–3.58)		2.37 (1.23–4.60)	
**Economic classification**								
A/B	1.00	< 0.001	1.00	0.021	1.00	< 0.001	1.00	0.019
C	2.04 (1.30–3.19)		1.81 (1.16–2.82)		1.94 (1.21–3.10)		1.71 (1.07–2.73)	
D/E	2.44 (1.54–3.86)		1.90 (1.20–3.00)		2.54 (1.57–4.09)		1.98 (1.22–3.19)	
**Occupation**								
Formal job	1.00	< 0.001	1.00	0.115	1.00	0.002	1.00	0.482
Informal job	0.81 (0.56–1.16)		0.72 (0.50–1.04)		1.10 (0.74–1.64)		1.00 (0.67–1.49)	
Retired	1.71 (1.13–2.59)		1.58 (0.96–2.60)		1.55 (0.95–2.53)		1.60 (0.88–2.88)	
Student	0.73 (0.40–1.32)		0.74 (0.39–1.41)		0.93 (0.50–1.73)		0.87 (0.46–1.65)	
Unemployed/housewife	1.42 (1.03–1.95)		1.00 (0.72–1.39)		1.73 (1.21–2.47)		1.19 (0.82–1.72)	
**Health status**								
Good	1.00	< 0.001	1.00	< 0.001	1.00	< 0.001	1.00	< 0.001
Fair	3.92 (3.01–5.11)		3.79 (2.90–4.96)		3.37 (2.55–4.45)		3.37 (2.56–4.44)	
Poor	9.64 (7.25–12.82)		9.06 (6.72–12.21)		9.02 (6.72–12.11)		8.99 (6.67–12.12)	

PR = prevalence ratios; CI = confidence interval.

**Figure 2:**
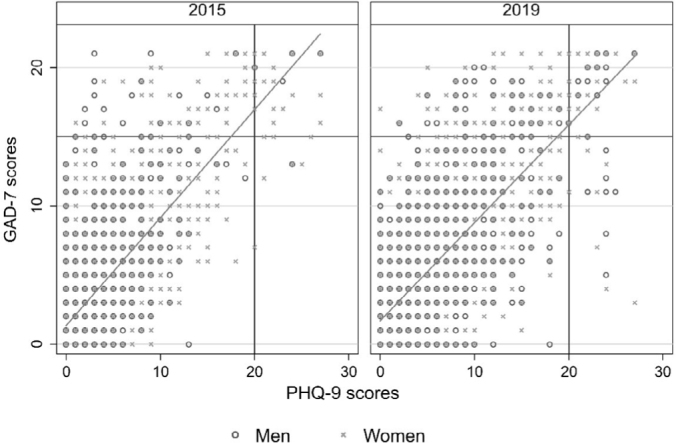
Depression and anxiety symptom scores from the Generalized Anxiety Disorder 7-item (GAD-7) and Patient Health Questionnaire 9-item (PHQ-9), for each year, according to sex.

Comparison between the 2019 and 2015 survey results revealed an increase in the prevalence of severe anxiety and depression symptoms, with a moderate to high correlation between the GAD-7 and PHQ-9 scores in 2015 (r = 0.726) and 2019 (r = 0.732; **Figure 2**).

Vertical and horizontal lines indicate the threshold for severe symptoms for each outcome.

## DISCUSSION

In 2019, the prevalence of severe anxiety and depression increased from 3% in 2015 to 9%. This increase was more pronounced in socioeconomically disadvantaged individuals, such as those with lower educational levels. The prevalence of severe anxiety and depressive symptoms was notably higher in 2019 among women, individuals with poor health status, and middle-class people. Pregnant women and informal workers had a lower prevalence of severe anxiety.

Our study was not primarily designed as a comparative analysis between the two surveys. The similar methodologies employed in each year enabled us to compare the changes in the study’s outcomes in these two distinct periods. Probabilistic sampling was adopted in both surveys to minimize selection bias, but all measurements were based on self-reports. Anxiety and depressive symptoms were assessed using two validated tools with strong psychometric properties that allow reliability when measuring these outcomes.^18,21^ Conservative cut-off points with higher sensitivity and specificity were used to determine the presence of anxiety and depressive symptoms.^20,24,25^ The present study reflects the status before the COVID-19 pandemic, which has since had a significant impact on mental health.^26^ Despite these limitations, our analysis provides valuable insights into the early effects of austerity measures on mental health in Manaus.

The prevalence of severe anxiety and depressive symptoms in our study, particularly in the 2019 survey, was higher than that reported in other countries. For example, a 2011–2014 study of 5,355 German adults reported a prevalence of severe anxiety symptoms of 4%.^27^ Similarly, an analysis of 13,829 adults living in Australia in 2020 showed a lower prevalence of severe depressive symptoms (4.5%).^28,29^ A representative survey from the United Kingdom (n = 17,152), conducted in 2014, reported severe depressive symptoms in only 3.3% of the population.^30^

The increase in the prevalence of severe anxiety and depressive symptoms was more pronounced among vulnerable individuals. In 2018, a cross-sectional study conducted in the United States with 22,682 adults found that financial concerns exacerbated mental health issues, particularly among the unemployed and low-income families due to daily exposure to stressors and the higher vulnerability of this group to stress.^31^

Our findings also indicate a higher prevalence of severe anxiety and depression in women than in men. Globally, women have consistently shown a higher prevalence and burden of depression and anxiety from 1990 to 2019.^32^ The psychosocial risk factors that can contribute to anxiety and depression are more frequent in women, such as domestic violence, gender harassment, employment and income inequalities, educational disparities, which increases stress.^33,34^

Symptom reporting is closely associated with the perception of poor health status, with a higher prevalence of symptoms correlating with greater dissatisfaction with one’s health conditions.^35^ Severe anxiety and depressive symptoms were higher among individuals with poorer self-reported health. A study of 1,241 patients from 28 primary care units in Spain in 2014–2017, using the same assessment tools as our research, found that anxiety and depressive symptoms were associated with lower quality of life.^36^ The overlapping diagnoses of mental disorders significantly worsens the quality of life.^37^ In a South Korean cohort including 1,204 community-dwelling older adults with anxiety and depression followed from 2001 to 2003, both conditions were associated with a higher incidence of comorbidities.^38^ The simultaneous presence of anxiety and depression exacerbated the physical disorders and disabilities after a 2-year follow-up period.^38^

Middle-class individuals exhibited a high prevalence of severe anxiety and depression. However, the poorest individuals had a higher probability of experiencing these conditions. A 2013 study conducted in 2,229 German adults found a significant correlation between socioeconomic status and clinically significant anxiety and severe anxiety.^39^ Another German cohort study that followed 12,484 adult individuals for 2.5 years identified socioeconomic status as a strong predictor of elevated depressive symptoms among individuals without these conditions at baseline.^40^ Depression and anxiety may also result in economic consequences and financial burdens. Individuals with lower socioeconomic status experience more depression- and anxiety-related absences from work.^41^ This mental distress can amplify social disadvantage, creating a vicious cycle where poorer individuals experience limited access to better employment opportunities and higher incomes, thus exacerbating the burden of mental disorders.^42^

Informal employment does not necessarily result in worse health outcomes but can expose underlying vulnerabilities.^43^ Informal workers have a lower prevalence of severe anxiety than formal workers. These results contrast with previous analyses, which suggest that informal work is associated with poorer health outcomes compared with formal work, particularly in low- and middle-income countries.^44,45^ A cross-sectional study of 8,680 non-agricultural workers from Spanish-speaking Central American countries, conducted in 2011, found a significant association between informal work and poor mental health.^46^ A previous analysis of the 2015 survey in the Manaus Metropolitan Region indicated that the health-related quality of life (measured using the European Quality of Life 5-Dimensions 3-Level instrument, which includes an anxiety/depression dimension) was lower among informal workers than among formal workers.^47^ The lack of labor and social protections may lead to poor working conditions, irregular income opportunities, barriers to healthcare access, and vulnerability to serious health shocks.^48^

## CONCLUSIONS

The prevalence of severe anxiety and depression symptoms tripled during the study period. The observed increase in mental distress may reflect the contextual changes, such as rising unemployment and difficulties in accessing health services. Positive variations in both outcomes were particularly pronounced among the most vulnerable individuals, underscoring the pivotal influence of social inequalities on the prevalence of mental disorders in the region. Ideally, further research should prospectively investigate the incidence of these outcomes and triggering factors that may increase mental distress in Manaus. Societal and health services improvements are also needed to address mental health comprehensively.
